# Assessment of Sprint Parameters in Top Speed Interval in 100 m Sprint—A Pilot Study Under Field Conditions

**DOI:** 10.3389/fspor.2021.689341

**Published:** 2021-06-21

**Authors:** Thomas Seidl, Tiago Guedes Russomanno, Michael Stöckl, Martin Lames

**Affiliations:** ^1^Chair for Performance Analysis and Computer Science in Sports, Faculty for Health and Sport Sciences, Technical University of Munich, Munich, Germany; ^2^Laboratory for Teaching Computer Science Applied to Physical Education and Sport, Faculty of Physical Education, University of Brasilia, Brasilia, Brazil; ^3^Institute of Sport Science, University of Vienna, Vienna, Austria

**Keywords:** sprint performance analysis, radio-based tracking, speed curve, top speed interval, intra-cyclic speed amplitude

## Abstract

Improving performances in sprinting requires feedback on sprint parameters such as step length and step time. However, these parameters from the top speed interval (TSI) are difficult to collect in a competition setting. Recent advances in tracking technology allows to provide positional data with high spatio-temporal resolution. This pilot study, therefore, aims to automatically obtain general sprint parameters, parameters characterizing, and derived from TSI from raw speed. In addition, we propose a method for obtaining the intra-cyclic speed amplitude in TSI. We analyzed 32 100 m-sprints of 7 male and 9 female athletes (18.9 ± 2.8 years; 100 m PB 10.55–12.41 s, respectively, 12.18–13.31 s). Spatio-temporal data was collected with a radio-based position detection system (RedFIR, Fraunhofer Institute, Germany). A general velocity curve was fitted to the overall speed curve (v_base_), TSI (upper quintile of v_base_ values) was determined and a cosine term was added to v_base_ within TSI (v_cycle_) to capture the cyclic nature of speed. This allowed to derive TSI parameters including TSI amplitude from the fitted parameters of the cosine term. Results showed good approximation for v_base_ (error: 5.0 ± 1.0%) and for v_cycle_ (2.0 ± 1.0%). For validation we compared spatio-temporal TSI parameters to criterion values from laser measurement (speed) and optoelectric systems (step time and step length) showing acceptable RMSEs for mean speed (0.08 m/s), for step time (0.004 s), and for step length (0.03 m). Top speed interval amplitude showed a significant difference between males (mean: 1.41 m/s) and females (mean: 0.71 m/s) and correlations showed its independence from other sprint parameters. Gender comparisons for validation revealed the expected differences. This pilot study investigated the feasibility of estimating sprint parameters from high-quality tracking data. The proposed method is independent of the data source and allows to automatically obtain general sprint parameters and TSI parameters, including TSI amplitude assessed here for the first time in a competition-like setting.

## Introduction

Improving performances in sprinting requires feedback on central sprint parameters ideally obtained in training as well as in competition. The 100 m-sprint can be divided into three main phases: acceleration phase, maximum velocity phase, and deceleration phase (Jones et al., 2009; Ae, 2017). In each phase, sprint parameters, such as split times, step length, step time, and ground contact time have been studied to improve the understanding of sprint performances and, subsequently, improve sprint performance based on those variables during training and competition (Moravec et al., 1988; Ae et al., 1992; Coh et al., 2001; Hunter et al., 2004; di Prampero et al., 2005; Morin et al., 2012; Colyer et al., 2018).

Since early velocity-time curve models proposed by Hill (1927) to very recent papers (Nagahara et al., 2018a, 2020; Bezodis et al., 2019; Morin et al., 2019; Ruiter and Van Dieen, 2019; von Lieres und Wilkau et al., 2020) research has focused on kinematic parameters of sprinting with a remarkable increase of papers analyzing the current world record of Usain Bolt in 2009 (9.58 s) (Beneke and Taylor, 2010; Taylor and Beneke, 2012; Krzysztof and Mero, 2013). However, there are still open questions such as the optimum relationship between step length and step time (Hunter et al., 2004; Bezodis et al., 2008; Debaere et al., 2013; Schubert et al., 2014).

Research has mostly investigated the first acceleration phase of the run (Nagahara et al., 2018a; Bezodis et al., 2019) typically trying to understand the step-to-step relations during acceleration based on ground reaction forces (Hunter et al., 2005; Rabita et al., 2015; Colyer et al., 2018; Nagahara et al., 2018a) and characteristic body angles by high-precision 3D kinematics (Manzer et al., 2016; Mattes et al., 2021).

The reason for the focus on this phase in sprint research may be found in the demanding methods of assessment employed (Mero, 1988; Nagahara et al., 2014a, 2019, 2020; Willwacher et al., 2016; Bezodis et al., 2019), which are typically, with only few exceptions (Nagahara et al., 2018a; Mattes et al., 2021), available only in laboratory settings. As it is a well-established fact that top speed is reached in 100 m-sprint only after around 40 m of maximum acceleration effort (Krzysztof and Mero, 2013; Healy et al., 2019), it is comprehensible that comparatively fewer studies exist which examined the maximum velocity phase or the final part of the acceleration phase. On the other hand, there is clear evidence that the maximum velocity phase is decisive for sprinting performance. Correlations between top speed and 100 m time are typically very high: *r* = 0.98 (Fuchs and Lames, 1990), *r* = 0.96 (Ryu et al., 2012), and *r* = 0.97 (Saito et al., 2008), making feedback on sprint parameters during this interval highly desirable.

Assessing sprint parameters in the maximum velocity phase in field settings either lack the necessary spatial or temporal resolution like timing gates, laser measurements, or manual video annotation (Brüggemann et al., 1999; Ferro et al., 2001; Graubner and Nixdorf, 2011; Krzysztof and Mero, 2013) or require an extensive instrumental setup such as motion capture systems or force plates applied at competitive and training tracks (Hunter et al., 2004; Park, 2011; Walker et al., 2019; Nagahara et al., 2020; Mattes et al., 2021). Hence, there are no satisfactory solutions for assessing sprint parameters in maximum velocity phase on a routine basis in competition and training.

However, due to the development of new tracking technologies with high spatial and temporal resolution (Sathyan et al., 2012; Seidl et al., 2016, 2017), like the radio-based tracking system RedFIR, (Grün et al., 2011) the analysis of sprint parameters in field settings has come into reach. As tracking systems are designed for routine analyses on training and competition sites they represent an excellent prospect for innovative contributions to sprint analysis and training.

Thus, the aims of the present study are two-fold. First, it aims at investigating the feasibility of using radio-based position detection for extracting sprint performance parameters including information on the maximum velocity phase in a field setting. Second, it proposes a data-driven method for obtaining the Top Speed Interval (TSI) and to derive TSI-specific sprint parameters including the intra-cyclic speed TSI amplitude, reported here for the first time. In addition, for validation the precision of the tracking data in this study and of the derived sprint parameters is explored, and comparisons between male and female sprinters as well as inter-correlations between sprint parameters are reported.

## Methods

### Sample and Data Acquisition

**Figure 1:**
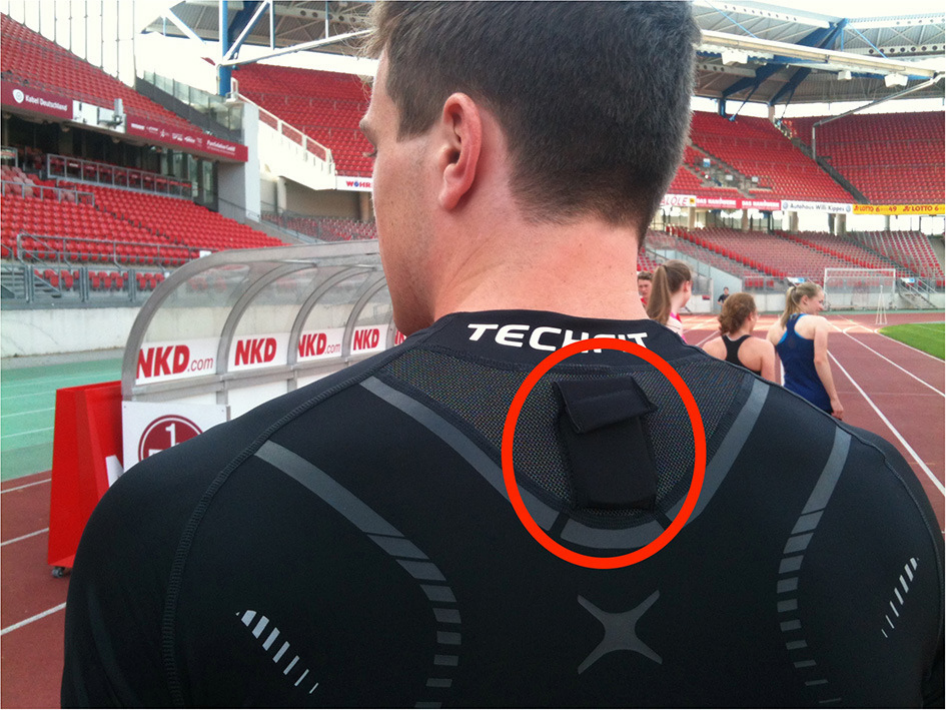
Transmitter placement on the athletes. Transmitters were placed inside a specially designed pocket.

We conducted our experiments in an official track and field stadium (Nuremberg, Germany), where a RedFIR radio-based tracking system (Fraunhofer Research Institute, Nuremberg, Germany) is installed. The RedFIR Real-Time Locating System (Grün et al., 2011) is based on time-of-flight measurements. Small transmitters (61 mm × 38 mm × 7 mm, 15 g) work with a sampling rate of 200Hz. Figure 1 shows the attachment of transmitters on the athletes' backs in a pocket of a compression shirt. For a more detailed description of the RedFIR system and the generated data streams see Grün et al. (2011), Mutschler et al. (2013), and Seidl et al. (2017).

Sixteen U20 athletes of regional and national level from surrounding clubs (age: 18.9 ± 2.8 years; IAAF points: 796 ± 146; specialization in 100 m) performed 32 100 m-sprints in total. Their personal bests ranged from 10.55 to 12.41 s (11.64 ± 0.68 s) for seven male and from 12.18 to 13.31 s (12.72 ± 0.43 s) for nine female sprinters, respectively. Each athlete performed two sprints in a competition-like setting after a 20 min warm-up that was chosen individually. Athletes rested at least 15 min between trials.

The study has been approved by the ethical committee of Technical University Munich and subjects gave informed consent.

### Data Analysis

A general assumption of this study is that there is an extended TSI in 100 m-sprint, where the sprinter runs at an almost constant, near-to-maximum or maximum speed. Further, it is assumed that running coordination is more or less stable in this interval (Brüggemann et al., 1999). We propose as operational definition of TSI as the upper quintile of all speed values over the full 100 m.

Based on these assumptions, we propose the following procedure to derive TSI parameters from raw tracking data that will be described in detail below:

(1) Fit a basic speed model *v*_*base*_(*t*) for 100 m sprint to raw speed data,
(2) Extract TSI,
(3) Fit a cyclic speed model to this interval to obtain TSI sprint parameters.

#### Basic Speed Model for Sprint Running

We start by applying a general sprint model that was proposed by Fuchs and Lames (1990), which is similar to one proposed by Arsac and Locatelli (2002), to obtain a *smooth* speed curve from raw speed data. In this model, running speed is perceived as the superposition of an acceleration and deceleration/fatigue process. Both processes are given by exponential growth functions:

$$\begin{array}{l} v_{base}\left(t\right)=A\left(1-\;e^{-\lambda t}\right)+B\left(1-e^{\mu t}\right), \end{array}$$

where *A* and *B* are weights for the corresponding acceleration/deceleration and λ, μ growth/decline rates, respectively.

**Figure 2:**
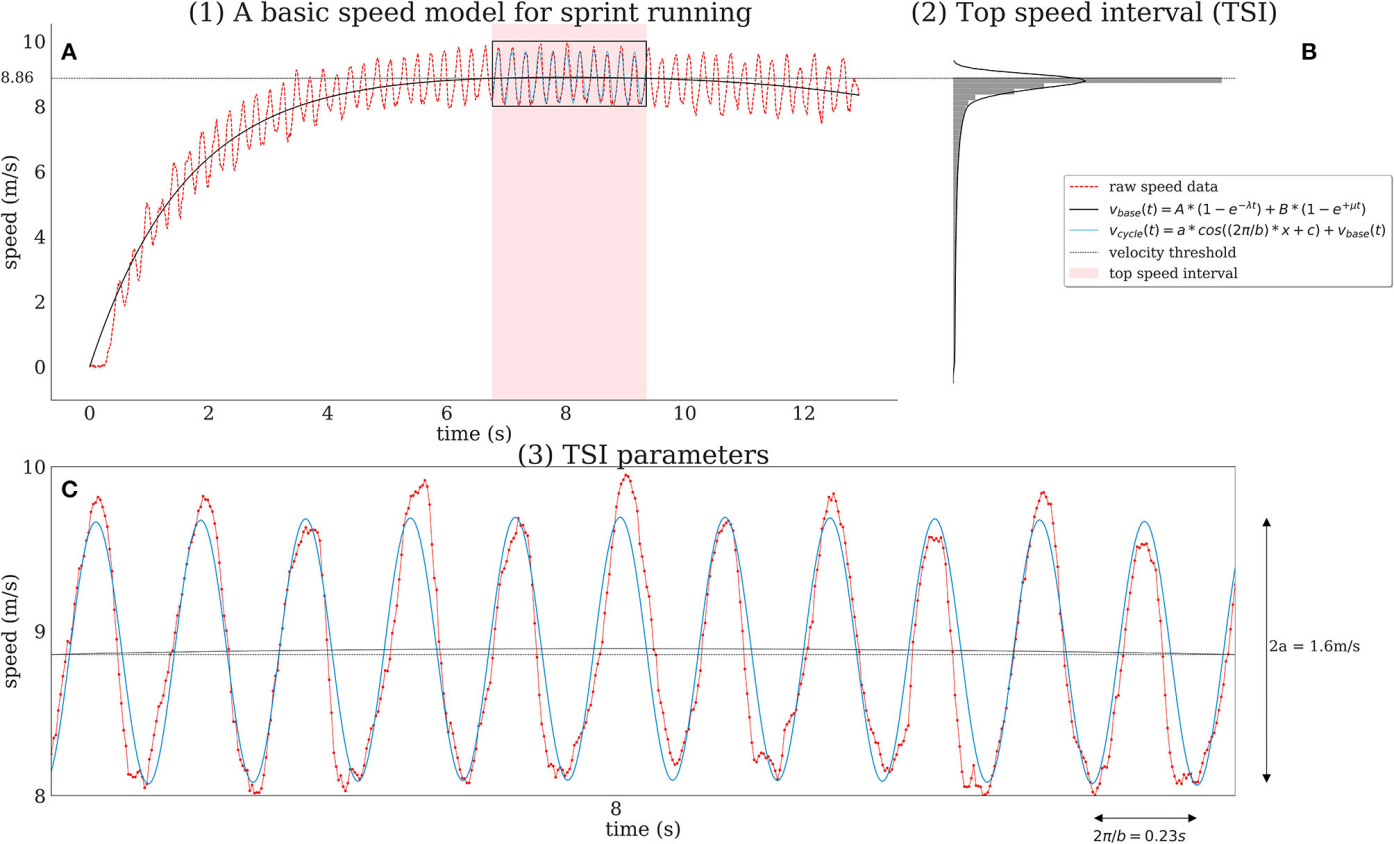
An overview of the three-step modeling process is shown for a sample run of a male sprinter. We start by fitting the basic speed model proposed by (Fuchs and Lames, 1990) **(A)**—application to raw data (red dotted line) yields a smooth speed curve (solid black line) and provides estimates for maximum speed, maximum acceleration, and speed endurance. We then obtain a run-specific Top Speed Interval [TSI, light red shaded area in **(A)**] which corresponds to the time period when running speed is within the top 20% percentile of an athlete's running speed for the given run **(B)**. Its calculation is based on the modeled speed (solid black line) rather than the raw speed data (dotted red line) and the corresponding velocity threshold (dotted black line, here: 8.86 m/s). Within the TSI we obtain intra-cyclic parameters (amplitude, step time, and step length) by fitting a cosine model (blue solid line) to raw speed data **(C)**.

Of course, any other smoothing method could be used as basic speed model if it validly describes the speed curve without the within-step velocity fluctuations (Bezodis et al., 2012). Applying the method from Fuchs and Lames (1990) allows for calculating the following sprint parameters: *maximum velocity* *v*_max_, *start acceleration* *a*_*start*_ and *speed endurance* *v*_*endu*_ given by the following equations:

$$\begin{array}{l} v_{\max}=v_{base}\left(t_{\max}\right)\;,\;t_{\max}=\{\begin{array}{c} \frac{\ln\left(\frac{A\lambda }{B\mu }\right)}{\lambda +\mu },\;B>0\\ t_{100},\;B=0 \end{array}\\ v_{endu}=\frac{v_{base}\left(t_{100}\;\right)}{v_{\max}},\\ a_{start}=a\left(0\right)=A\lambda -B\mu \end{array}$$

where *a*(*t*) is the *acceleration* [derivation of *v*_*base*_(*t*)]

$$\begin{array}{l} a\left(t;A,B,\lambda ,\mu \right)=A\lambda e^{-\lambda t}-B\mu e^{\mu t} \end{array}$$

and *t*_100_ is the time for 100 m obtained from tracking data. *Speed endurance* *v*_*endu*_ is a measure of the ability of athletes to maintain their top speed and is calculated relative to top speed *v*_max_. Hence, *v*_*endu*_ will be 1 if top speed is maintained until the finish line (Bompa and Bompa, 1999). An example of raw data and fitted base model is depicted in Figure 2A.

#### Extract TSI

Sprint steps in the TSI are supposed to show a stable (top speed) coordination pattern (Brüggemann and Rühl, 1990). We define TSI to be the time interval when athletes are running at a speed within the top quintile of their speed distribution over the full 100 m track, as shown in Figure 2B:

$$\begin{array}{l} TSI:=\left\{t\;|\;v\geq Q_{4},\;v\in v_{base}\;\right\}, \end{array}$$

where *Q*_4_ is the lower border of the top quintile of speed values from function *v*_*base*_. This threshold was chosen by convention to make sure TSI contains “enough” cycles for a stable parameter estimation in the next step and still meeting the requirement of constant coordination patterns.

#### TSI Parameters

To capture the cyclic structure of the speed within TSI we propose a cosine function added to *v*_*base*_. The model function for TSI then is:

$$v_{cycle}\left(t\right)={\text{a}}^{*}\text{cos}\left(2\pi /bt+c\right)+v_{base}\left(t\right),$$

where 2π/*b* estimates *top speed step time*. Analogously, we obtain *top speed step length* by performing the calculations in the distance domain instead of time domain. *c* is an offset/shift parameter and *v*_*base*_(*t*) corresponds to the function value of the base speed model during TSI. *a* is the amplitude of the cosine, i.e., 2a is the *intra-cyclic top speed amplitude* which is defined as the maximum speed difference between two consecutive ground contacts in the TSI (Figure 2C).

Sprint time is measured by the tracking system (sprint time tracking) thus not including reaction time. A summary of obtained variables is shown in Table 1.

**Table 1:** Overview for automatically derived parameters.

**Parameters**	**Unit**	**Definition**	**Source**
Sprint time	(s)	Sprint time measured by the RedFIR system (no reaction time)	Tracking system
Max. speed	(m/s)	Maximum speed derived from v_base(t)	v_base
Start acceleration	(m/s^2^)	Maximum acceleration derived from v_base(t)	v_base
Speed endurance	(%)	Speed endurance derived from v_base(t)	v_base
Top speed start location	(m)	Start position in running direction (m) of the top speed interval based on threshold obtained by top quintile of speed distribution for the run	v_base
Top speed end location	(m)	End position in running direction (m) of the top speed interval based on threshold obtained by top quintile of speed distribution for the run	v_base
Top speed length	(m)	Distance covered (m) within top speed interval based on threshold obtained by top quintile of speed distribution for the run	v_base
Top speed start time	(s)	Start time (s) of the top speed interval based on threshold obtained by top quintile of speed distribution for the run	v_base
Top speed end time	(s)	End time (s) of the top speed interval based on threshold obtained by top quintile of speed distribution for the run	v_base
Top speed duration	(s)	Duration (s) of the top speed interval based on threshold obtained by top quintile of speed distribution for the run	v_base
Top speed step time	(s)	Mean step time during top speed interval	v_cycle
Top speed step length	(m)	Mean step length during top speed interval derived	v_cycle
Top speed amplitude	(m/s)	Peak-to-peak amplitude of intracyclic speed during top speed interval	v_cycle
Normalized top speed amplitude	(%)	Peak-to-peak amplitude of intracyclic speed during top speed interval—normalized by maximum speed	v_cycle

*Units, definitions, and source for each parameter is shown. E.g., maximum speed is derived from v_base_(t) whereas TSI amplitude is derived from v_cycle_(t)*.

For each run, we fitted the curves *v*_*base*_ and *v*_*cycle*_ by solving the respective least squares optimization problem (Levenberg, 1944; Marquardt, 1963). Data analysis has been done using Python 3.7 and scipy package.

### Validation of RedFIR for Estimating TSI Sprint Parameters

The RedFIR system as such has been validated before in a sprint-specific study (Seidl et al., 2017). To assess the validity of derived top speed parameters based on the proposed method we conducted additional validation experiments in the same location by simultaneously recording 100 m-sprints with RedFIR and criterion systems for horizontal speed (Laveg −13 runs) and step parameters (OptoGait −23 runs).

A Laveg laser (Jenoptik, Germany; 100 Hz) was positioned 13 m behind the starting line and an operator aimed the laser on the spot on the back between the shoulder blades where the RedFIR transmitter was located. The Laveg system is known to provide accurate and reliable estimates for displacement and speed (Harrison et al., 2005). Criterion speed was derived from Laveg distance data by fitting a fifth-order polynomial to positions and analytical differentiation afterwards (Bezodis et al., 2012). For each run the location of TSI was estimated based on our method on RedFIR data, yielding start and end locations of TSI on the 100 m track, e.g., (54 m, 73 m). We then calculated the mean speed based on Laveg speed at the same section of the track and compared it to the results obtained by RedFIR.

Simultaneously, a photoelectric measurement system OptoGait (Microgate, Bolzano, Italy/OJ) was placed to cover the second half of the 100 m track (50–100 m) providing step-by-step estimates for step length and step time. The OptoGait system is comprised of 1 m modules, which can be attached to each other to cover a larger distance. Each bar was 100 × 8 cm and contained 96 light diodes that were located 3 mm above floor level and approximately 1 cm apart. Lienhard et al. (2013) reported 95% limits of agreement of 1.0–1.8 cm for step length, 0.007–0.023 s for cycle time for older adults walking. Although we are using the OptoGait system as a criterion for analyzing sprint parameters obtained from young sprinters we deem this system to be a valid choice as our approach only provides mean values for step length and step time within TSI. OptoGait is often used as criterion system for evaluation studies in sprinting (Gindre et al., 2016; Schmidt et al., 2016). For more details on the validation setting see Seidl et al. (2017). Mean step length and step time estimates within the TSI based on RedFIR data were compared to criterion measurements captured by the OptoGait system.

### Statistical Analysis

All statistical analyses were performed in SPSS, Version 25.0 (SPSS, Inc., Chicago, IL, USA). The level of significance was set at *p* = 0.05 and descriptive results were expressed as mean ± *SD*. Variables were checked for normality using the Kolmogorov-Smirnov and Shapiro-Wilk's test. For normally distributed parameters a *t*-test for independent samples was used to investigate differences between male and females. For variables TSI start location, TSI end location, TSI amplitude, and normalized TSI amplitude non-normal distribution were observed and, hence, Mann-Whitney's U-test was used.

Effect size was calculated and classified according to Cohen's classification of effect sizes into small (*d* ≤ 0.2), moderate (*d* ≤ 0.5), and large (*d* ≥ 0.8) effects (Cohen, 1988).

The relationships between sprint parameters (independent variables) and sprint time (dependent variable) were investigated by calculating Spearman correlations coefficients (small effect <0.3; medium <0.5; large >0.5). Reliability of sprint variables was assessed calculating intra-class correlation coefficients (ICC, two-way mixed methods, single measurements, absolute agreement). ICC coefficients were classified according to Koo and Li (2016) into poor (ICC ≤ 0.5), moderate (ICC ≤ 0.75), good (ICC ≤ 0.9), and excellent (ICC > 0.9).

## Results

### Validation of TSI Parameters

Mean speed differences (RedFIR—criterion) within TSI were −0.03 m/s whereas RMSE was 0.08 m/s (1.05%). Errors for mean step time (step length) were −0.001 s (0.001 m) showing a slight underestimation of the RedFIR system with a RMSE for step time of 0.004 s (1.67%) and 0.03 m (1.43%) for step length showing a slight overestimation, respectively. Top speed parameter estimates for mean speed, mean step length, mean step time for the radio-based tracking, and criterion systems are shown in Table 2. Bland-Altman plots are shown in the Supplementary Material.

**Table 2:** Validation results when comparing TSI mean speed, TSI mean step time, and TSI mean step length derived from ther RedFIR system to criterion measurements for speed (Laveg laser), step time (OptoGait), and step length (OptoGait).

		Criterion^*^				**RedFIR**				**Differences (RedFIR – Criterion)**						
**Parameter**	***n***	**Mean**	**Std**	**Min**	**Max**	**Mean**	**Std**	**Min**	**Max**	**Mean**	**Mean abs**	**Percentage abs**	**RMSE**	**Percentage RMSE**	**loa**	**Percentage loa**
Mean speed (TSI) (m/s)	14	7.700	0.560	7.010	8.930	7.660	0.580	6.990	8.960	−0.040	0.060	0.010	0.085	0.011	[−0.19, 0.10]	[−0.02, 0.01]
Mean step time (TSI) (s)	23	0.250	0.010	0.229	0.273	0.249	0.008	0.236	0.262	−0.001	0.003	0.012	0.004	0.017	[−0.05, 0.06]	[−0.03, 0.03]
Mean step length (TSI) (m)	23	1.954	0.150	1.630	2.230	1.955	0.150	1.660	2.220	0.001	0.020	0.012	0.027	0.014	[−0.01, 0.01]	[−0.04, 0.03]

*All variables are captured with a percentage RMSE of <2%*.

* *Laveg for speed/OptoGait for step time and step length*.

### Results for TSI Parameters

The method was successfully applied to all 32 trials and giving RMSE as percentage of maximum speed showed a good fit for *v*_*base*_(*t*) (5.0 ± 1.0%) as well as for *v*_*cycle*_(*t*) (2.0 ± 1.0%).

**Figure 3:**
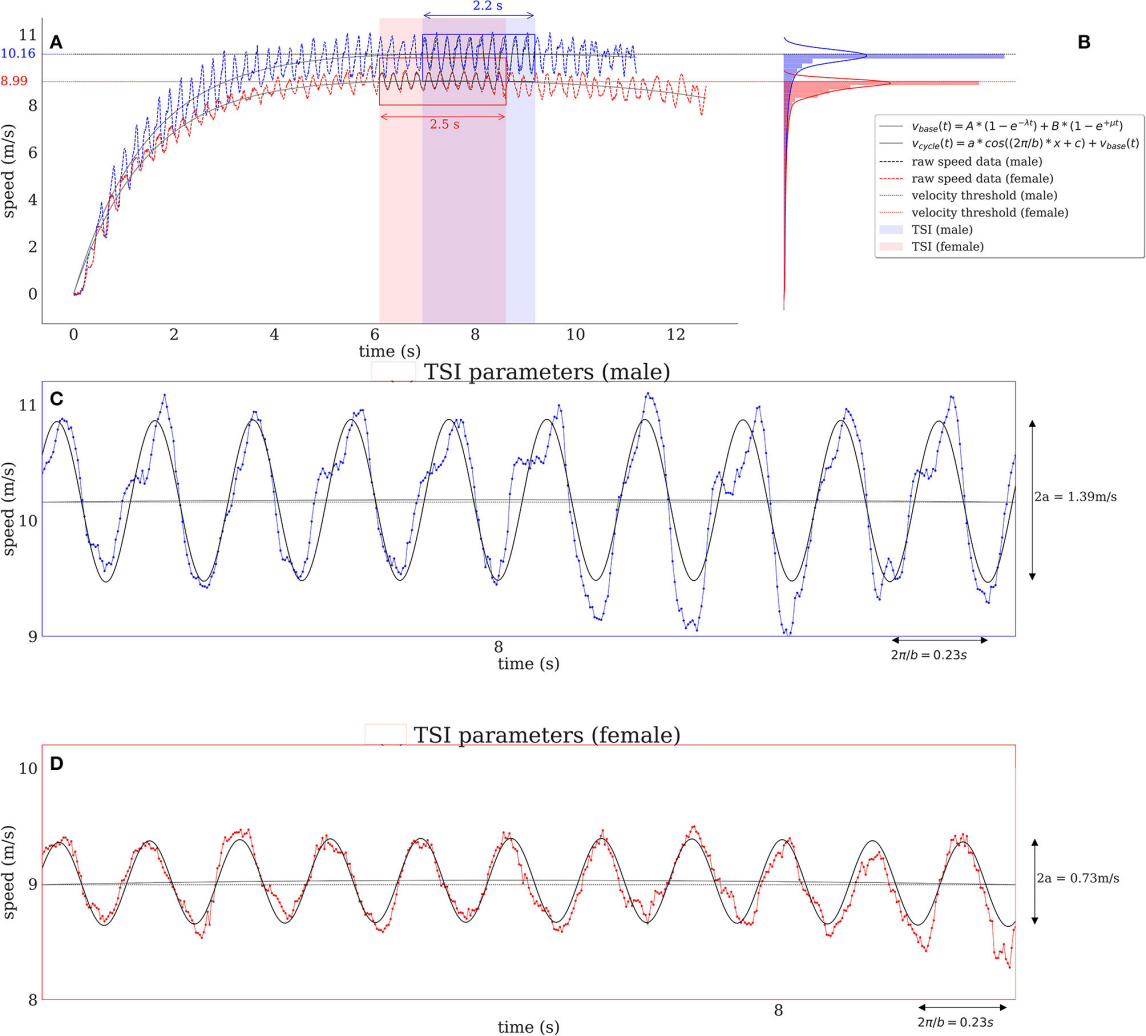
Comparison for two runs performed by female (red) and male (blue) athletes. Panel **(A)** shows raw speed (dotted line) and fitted *v*_*base*_(*t*) which allows to estimate TSI start and end time for each run individually by estimating a run-specific speed threshold based on the observed speed distribution **(B)**. Zoomed views of TSI are shown for a male **(C)** and a female runner **(D)**. Within TSI we obtain TSI step time (which is the same for both runners −0.23 s) and TSI amplitude which is larger for the male runner (1.39 vs. 0.73 m/s).

A graphical description of the best male and female performance is given in Figure 3. It shows the clear cyclical structure of running speed and its rather constant pattern in TSI allowing for fitting our cyclic TSI-speed model. The faster male runner reaches TSI later with a short duration but at a higher maximum speed compared to the female sprinter. Within TSI both show the same step time (0.23 s) but different TSI speed amplitude (1.39 vs. 0.73 m/s).

Table 3 shows descriptive statistics for TSI parameters. Reliability tests for TSI parameters showed good to excellent reliability except for speed endurance, TSI start and end time, top speed distance, TSI start, and end location.

**Table 3:** Descriptive statistics for sprint time and obtained sprint parameter values for 18 female and 14 male runs.

	**Male**				**Female**				***t*****-test**			**Reliability ICC**
	**Mean**	**Std**	**Min**	**Max**	**Mean**	**Std**	**Min**	**Max**	***t*-Values**	***p*-Values**	**Effect size**	
Sprint time (s)	11.93	0.56	11.21	12.67	13.08	0.44	12.34	14.11	6.47	*p* < 0.001	2.22	0.963
Max. speed (m/s)	9.44	0.50	8.86	10.18	8.47	0.35	7.88	9.03	−6.23	*p* < 0.001	2.26	0.990
Start acceleration (m/s^2^)	6.29	0.55	5.39	7.32	5.84	0.44	4.92	6.57	−2.56	*p* < 0.016	0.90	0.851
Speed endurance (%)	93.11	3.23	88.07	98.54	91.06	3.69	84.01	97.99	−1.65	*p* < 0.109	0.59	0.190
Top speed start location (m)	47.94	4.82	43.01	59.06	43.08	4.51	36.17	57.08	210.00	*p* < 0.001	0.56	0.440^+^
Top speed end location (m)	68.64	4.80	63.53	79.39	63.92	4.70	57.69	78.63	203.00	*p* < 0.003	0.51	0.416^+^
Top speed length (m)	20.69	0.66	19.96	22.27	20.84	0.68	19.73	22.04	0.60	*p* < 0.552	0.21	0.336
Top speed start time (s)	6.52	0.60	5.76	7.93	6.47	0.59	5.54	8.11	−0.25	*p* < 0.802	0.09	0.424
Top speed end time (s)	8.72	0.65	7.83	10.18	8.93	0.61	8.12	10.55	0.94	*p* < 0.357	0.33	0.518
Top speed duration (s)	2.20	0.13	2.01	2.41	2.47	0.12	2.30	2.68	5.83	*p* < 0.001	2.07	0.903
Top speed step time (s)	0.23	0.01	0.22	0.24	0.24	0.01	0.22	0.26	1.51	*p* < 0.141	0.54	0.914
Top speed step length (m)	2.10	0.09	1.99	2.21	2.00	0.09	1.80	2.14	−2.97	*p* < 0.006	1.06	0.865
Top speed amplitude (m/s)	1.31	0.31	0.82	1.77	0.71	0.11	0.51	0.91	245.00	*p* < 0.001	0.80	0.976
Normalized top speed amplitude (%)	13.88	3.18	8.93	18.27	8.42	1.25	6.26	10.40	227.00	*p* < 0.001	0.67	0.970

*Sprint time was measured by the RedFIR system. Maximum speed, maximum acceleration and speed endurance are obtained by v_base_(t) whereas amplitude, step length and step time are obtained by v_base_(t). Results of t-tests/Mann-Whitney (t-value, p-value and effect size d)*.

+ *Mann Whitney U-test*.

Comparison of TSI parameters between male and female sprinters showed significant differences for maximum speed, TSI start location, and TSI end location. For females mean TSI speed amplitudes were 0.71 m/s, which corresponds to 8.42% of respective maximum speed. For males we even found TSI speed amplitudes of 1.31 m/s (13.88% of max speed). Top speed interval speed amplitudes of male runners were, on average, 0.60 m/s (5.4%) larger than for females (*U* = 245, *p* < 0.001, *d* = 0.8). This was also the case for TSI amplitude normalized with maximum speed (*U* = 227, *p* < 0.001, *d* = 0.67).

Table 4 shows correlations between sprint parameters for male and female sprinters that give an insight into the respective structures of sprinting performance.

**Table 4:** Correlation Matrix separately for male (lower triangular matrix, white) and females (upper triangular matrix, gray).

	**Male**													
	**Sprint time (s)**	**Max. speed (m/s)**	**Start acceleration (m/s^**2**^)**	**Speed endurance (%)**	**Top speed start location (m)**	**Top speed end location (m)**	**Top speed length (m)**	**Top speed start time (s)**	**Top speed end time (s)**	**Top speed duration (s)**	**Top speed step time (s)**	**Top speed step length (m)**	**Top speed amplitude (m/s)**	**Normalized top speed amplitude (%)**
Sprint time (s)	1.000	−0.912^**^	−0.798^**^	0.138	0.125	0.116	−0.218	0.420	0.695^**^	0.808^**^	0.218	0.323	−0.248	0.090
Max. speed (m/s)	−0.864^**^	1.000	0.749^**^	−0.121	−0.073	−0.068	0.279	−0.402	−0.682^**^	−0.795^**^	−0.073	−0.121	0.169	−0.156
Start acceleration (m/s^2^)	−0.176	0.083	1.000	0.090	−0.182	−0.152	0.367	−0.429	−0.609^*^	−0.570^*^	−0.068	0.108	0.196	−0.059
Speed endurance (%)	−0.265	0.014	−0.465	1.000	0.824^**^	0.829^**^	0.134	0.732^**^	0.629^*^	0.073	−0.521	−0.160	−0.103	−0.042
Top speed start location (m)	−0.408	0.355	−0.659^**^	0.834^**^	1.000	0.987^**^	0.103	0.881^**^	0.706^**^	0.011	−0.459	−0.415	−0.143	−0.077
Top speed end location (m)	−0.378	0.316	−0.612^**^	0.864^**^	0.969^**^	1.000	0.204	0.868^**^	0.715^**^	0.075	−0.455	−0.393	−0.103	−0.046
Top speed length (m)	−0.076	−0.018	0.148	0.340	0.153	0.317	1.000	0.002	−0.009	0.289	0.332	−0.020	−0.011	0.033
Top speed start time (s)	−0.073	0.009	−0.827^**^	0.839^**^	0.904^**^	0.892^**^	0.112	1.000	0.915^**^	0.267	−0.363	−0.354	−0.314	−0.160
Top speed end time (s)	0.067	−0.195	−0.764^**^	0.849^**^	0.786^**^	0.809^**^	0.278	0.941^**^	1.000	0.593^*^	−0.233	−0.156	−0.295	−0.044
Top speed duration (s)	0.623^**^	−0.782^**^	0.028	0.202	−0.177	−0.059	0.596^**^	0.069	0.327	1.000	0.374	0.242	−0.123	0.218
Top speed step time (s)	0.033	−0.093	−0.434	0.230	0.255	0.207	−0.181	0.402	0.411	0.102	1.000	0.516	−0.182	−0.420
Top speed step length (m)	−0.557^*^	0.710^**^	−0.412	0.230	0.589^*^	0.498^*^	−0.183	0.428	0.233	−0.606^**^	0.501^*^	1.000	−0.182	−0.156
Top speed amplitude (m/s)	−0.163	0.371	−0.189	−0.102	0.232	0.108	−0.321	0.180	0.015	−0.443	0.259	0.575^*^	1.000	0.912^**^
Normalized top speed amplitude (%)	0.038	0.192	−0.121	−0.284	0.022	0.106	−0.424	0.035	−0.100	−0.356	0.259	0.416	0.959^**^	1.000

*Sprint time corresponds to the time it took an athlete to cover the distance between start and finish and was obtained directly from the tracking system. For male athletes top speed step length shows significant correlations with top speed step time (r = 0.50), maximum speed (r = 0.71), TSI amplitude (r = 0.58), top speed duration, top speed start location (r = 0.59), top speed end location (r = 0.50), sprint time tracking (r = −0.56) and sprint time stopwatch (r = −0.57) In contrast, for females top speed step length is not correlated with other sprint variables*.

* *p < 0.05*,

** *p < 0.01*.

There were several significant correlations concerning TSI and other sprint parameters, e.g., TSI start and end location correlated significantly with speed endurance (male: *r* = 0.824, *p* < 0.01; *r* = 0.829, *p* < 0.01; female: *r* = 0.834, *p* < 0.01; *r* = 0.864, *p* < 0.01). TSI time parameters and location parameters showed similar results.

Top speed amplitude (absolute and % of max speed) did not show a significant correlation to any other sprint parameter (exception TSI step length in females: *r* = 0.575, *p* < 0.05) indicating that it is a sprint parameter independent of all other sprint determinants.

## Discussion

The study was in first place aiming at investigating the appropriateness of radar-based position detection as a routine instrument for obtaining sprint parameters in competition and training. This is motivated by our perception that especially for sprint parameters related to the TSI there is a considerable lack of routine diagnostics of sprint parameters despite the decisive importance of TSI for the 100 m-sprint.

The measurement device, the RedFIR system, was validated for our study in comparison to laser-based speed measurements and step length and step time obtained from Optogait. Regrettably, there was a mismatch between Optogait measurement interval (50–100 m) and TSI (start typically <50 m). Nevertheless, the deviations of RedFIR-based measurements were acceptable (speed: 1.05%, step time: 1.67%, step length: 1.43%). In addition, a direct validation of the TSI speed amplitude for example with a high precision, marker based optical tracking system would be desirable.

The suggested model for identifying TSI is solely based on investigating the horizontal speed distribution of the full 100 m sprint. This circumvents the need for estimating the COM from a full body model (von Lieres und Wilkau et al., 2020) and/or the analysis of consecutive step characteristics derived from motion capture data (Nagahara et al., 2014a). We used the upper quintile of the overall speed values obtained. This threshold implies though, that the speed curve enters and leaves TSI only with a small speed slope thus leading to a lack of reproducibility (ICC for TSI start and end location/time between 0.336 and 0.518). Maybe a lower threshold could cure this but one would have to be still sure, that the important assumption of a stable top speed running pattern is not violated. Thus, it would be beneficial to investigate the optimal percentile for defining the TSI in subsequent studies.

The procedure of identifying TSI parameters is quite straightforward. The regression fitting errors for v_base_ (5.0 ± 1.0%) and v_cycle_ (2.0 ± 1.0%) were acceptable.

The suggested method for obtaining TSI sprint parameters fits a cyclic sprint speed model to each sprint step in TSI. As a “by-product” an estimation of the intra-cyclic speed amplitude in TSI is obtained. To the best of the authors' knowledge this is the first time this parameter is explicitly reported when calculating sprint parameters for TSI. This is somewhat surprising, because there is some evidence that TSI speed amplitudes do exists. If only positional data with a sufficiently high frequency (≥200 Hz) and high spatial accuracy are available, intra-cyclic speed variations are readily observed for example when tracking sprinters with a laser device, e.g., in the documentation of Usain Bolt's world record these cyclic patterns are obviously present (Graubner and Nixdorf, 2011).

Coh et al. (2018) report a similar phenomenon in their analysis of a 100 m event at Zagreb in 2011 (including Usain Bolt running 9.85 s). They examined the ground contacts in his fastest 20 m interval (70–90 m) and found a minimal speed of 11.13 m/s and a maximum of 12.04 m/s at toe-off resulting in an intra-contact speed amplitude for the horizontal displacement of COM of 0.91 m/s (this corresponds to 7.5% standardized amplitude). Notwithstanding some criticism on the applied methodology (2D-kinematics from 100 Hz videos with pixel resolution of 720 × 576) and the fact that this is intra-contact speed, these results are evidence for intra-step speed variation.

Also, there is a considerable body of studies on horizontal braking and acceleration impulses during ground contact in sprinting (Nagahara et al., 2014b, 2018a,b). These are large braking and accelerating impulses and only the small difference between them may be used for propulsion. These impulses impact on the whole body and must give rise to cyclic intra-step speed variation, although to the best of our knowledge this consequence was not mentioned in the quoted papers.

It must be assumed, though, that the absolute values for intra-cyclic speed amplitudes depend on the body part of sensor fixation. Linke et al. (2018) report significant differences between measurements of running kinematics taken from center of shoulders like in our study and center of pelvis, with the latter representing center of mass better. It is not yet well-understood how braking and acceleration impulses from ground contact of sprinters propagate through the body giving rise to intra-cyclic speed variations. Theoretically, there is constant speed within flight time and the only changes of speed may be induced in contact time. Nevertheless, properties of the human body (body segments with non-rigid connections) may care for “dampening” of the net ground reaction forces in higher body locations, e.g., center of mass, and center of shoulders, where our sensors were placed. The goodness-of-fit results of the cosine-wave model and the qualitative shape of within-cycle speed support this notion, but as mentioned there is reason to assume that TSI amplitude is specific to the investigated body part, e.g., center of shoulders (examined here) or center of pelvis or even center of mass.

The absolute values for the amplitude of the cyclic component in sprint speed in TSI for the center of shoulders are quite high (mean females: 0.71 m/s; 8.42% of maximum speed; mean males 1.31 m/s; 13.88%). This means, within one step a sprinter is exposed to considerable accelerations and decelerations. The question that suggests itself is why these are hardly perceived and reported by the athletes. A first speculation could take into account that, because there are braking and acceleration impulses in normal gait also, we have learnt by assimilation (Piaget and Inhelder, 1967) and habituation (Hinde, 1970) in early childhood to ignore this irrelevant sensor input. Thus, even a top-level sprinter might adhere to a perception of constant running speed.

Nevertheless, it might turn out that intra-cyclic amplitude will become a valuable parameter for sprint diagnostics. Coh et al. (2018) make a low braking force responsible for Usain Bolt's high sprinting speed. This would correspond to a smaller TSI amplitude, although there should be a mechanical lower limit for it. It may be expected that there is an individual optimum for TSI amplitude as too large values should be too energy demanding and too small ones do not allow generating sufficient propulsion forces.

Descriptive statistics and gender comparisons allow a deeper understanding of TSI. Female sprinters stay a significantly longer time in TSI due to reaching it earlier and due to mostly leaving it later. There are no significant differences in the length of TSI; female sprinters reach and leave it after significantly shorter distances. It must be mentioned that this study was designed as a technical feasibility study and not for gender comparisons.

The same holds for the correlations reported in this research. These were mainly included to show the construct validation of the performance structure of the 100 m sprint. The inter-correlations between the sprint parameters confirm the decisive role of maximum speed for the 100 m performance with *r* = −0.912 male/−0.864 female. Maximum speed itself is influenced negatively by the duration and end time (not for females) of TSI as well as acceleration at start.

The TSI parameters for location correlate with sprint endurance: the farther TSI is down the way the smaller the loss of speed toward the end. The same holds for the temporal onset and offset of TSI. Step time and step length in TSI show low to moderate correlations with other sprint parameters for the male athletes, whereas for female athletes we find significant relationships to other TSI parameters as well as to maximum speed. The correlation between step length and time is moderate (*r* = 0.516 male/0.501 female). This could be explained by a combination of step length and time that constitutes an individually optimal relation. This relation is determined by individual muscular and anthropometric properties (Salo et al., 2011; Van Oeveren et al., 2019).

Finally, there were no significant correlations found for the intra-cyclic speed amplitude for the male athletes and only with top speed step length for female athletes. This could mean that the top speed amplitude constitutes a rather independent dimension of sprint performance impacting maximum speed with *r* = 0.370 (male only 0.169).

The aims of the study were to demonstrate that employing sensor-based position detection allows for comprehensive sprint diagnostics in competition and training settings. It must be mentioned that the sprint parameters obtained do not rely on the specific technological platform, here the RedFIR system. Instead, any platform providing sufficiently accurate high frequency position data from sprinters may be used. The increasing market for sensor-based position detection in sports, mostly powered by the demand of professional team sports, will make appropriate systems much more available in the future.

As it is a well-known fact that top speed is decisive for the overall performance in 100 m-sprint, a method assessing sprint parameters like TSI step length and step time are of high relevance. The newly introduced parameter TSI amplitude also bears potential for improving the theoretical understanding as well as training in top level sprint athletes.

## Data Availability Statement

The datasets presented in this article are not publicly available. Requests to access the datasets should be directed to martin.lames@tum.de.

## Ethics Statement

The studies involving human participants were reviewed and approved by Technical University of Munich. Written informed consent from the participants' legal guardian/next of kin was not required to participate in this study in accordance with the national legislation and the institutional requirements.

## Author Contributions

All authors listed have made a substantial, direct and intellectual contribution to the work, and approved it for publication.

## Conflict of Interest

The authors declare that the research was conducted in the absence of any commercial or financial relationships that could be construed as a potential conflict of interest.

## Supplementary Material

Supplementary Figure 1 Bland-Altman plots for TSI speed vs. Laveg (left), TSI step length (middle), and TSI step time vs. OptoGait, respectively. Systematic differences (solid line) and 95% limits of agreement (dashed lines) are shown.
