# Supplementary figures and images for: Assessment of Sprint Parameters in Top Speed Interval in 100 m Sprint—A Pilot Study Under Field Conditions
Source: Front Sports Act Living. 2021 Jun 21;3:689341. doi: 10.3389/fspor.2021.689341 (PMC8255486; doi:10.3389/fspor.2021.689341)

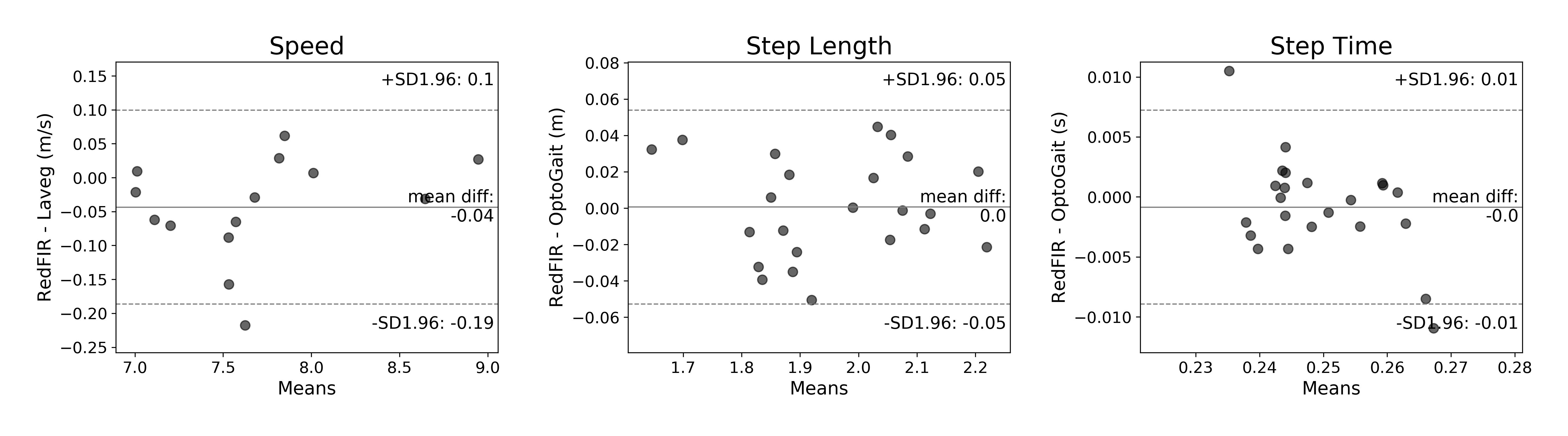

Supplement: Supplementary Figure 1 — Bland-Altman plots for TSI speed vs. Laveg (left), TSI step length (middle), and TSI step time vs. OptoGait, respectively. Systematic differences (solid line) and 95% limits of agreement (dashed lines) are shown. [file Image_1.PNG]
